# A road for a promising future for China’s primates: The potential for restoration

**DOI:** 10.24272/j.issn.2095-8137.2018.032

**Published:** 2018-05-12

**Authors:** Colin A. Chapman

**Affiliations:** 1 Department of Anthropology, McGill University, Montréal Québec H3A 2T7, Canada; 2 Wildlife Conservation Society, Bronx New York 10460, USA; 3 Section of Social Systems Evolution, Primate Research Institute, Kyoto University, Inuyama 484-8506, Japan

**Keywords:** Conservation, Forest restoration, Regeneration, Primate population dynamics

## Abstract

China is one of the most dynamic countries of the world and it shelters some amazing levels of biodiversity, including some very special primate species. However, primarily as a result of forest loss, most of which occurred in historical times, approximately 70% of China’s primate species have less than 3 000 individuals. Here I evaluate one road for future conservation/development that could produce very positive gains for China’s primates; namely forest restoration. I argue that for a large scale restoration project to be possible two conditions must be met; the right societal conditions must exist and the right knowledge must be in hand. This evaluation suggests that the restoration of native forest to support many of China’s primates holds great potential to advance conservation goals and to promote primate population recovery.

## INTRODUCTION

The world is changing rapidly and China represents one of the most dynamic countries on earth and it shelters some amazing levels of biodiversity (i.e., >30 000 species of vascular plants (behind only Brazil and Colombia), ~2 340 species of terrestrial vertebrates (Liu et al., 2003)). Globally, biodiversity is being lost at an accelerating rate, with current extinction rates approximately 1 000 times higher than background rates (Pimm et al., 2014). Recent estimates suggest that 11 000–58 000 species are lost each year and that surviving vertebrate species have declined in abundance by 25% since 1970 (Dirzo et al., 2014). Humans are clearly responsible for this accelerating loss of biodiversity, including the endangerment of primates. Between 2000 and 2012, 2.3 million km${}^{2}$ of forest was lost globally and in the tropics forest loss increased each year (Hansen et al., 2013). To put this in perspective, this area is approximately the size of Mexico. Global estimates of the extent of wildlife over-exploitation are very poor. However, Bennett et al. (2000) estimated that six million mammals were hunted annually in Malaysian Borneo. With respect to climate change, temperatures are predicted to increase by 1.5 ${}^{\circ }$C by the end of the 21st century (IPCC, 2014) and using moderate greenhouse gas emission estimates, it is projected that by 2100 75% of all tropical forests present in 2000 will experience temperatures that are higher than the temperatures presently supporting closed canopy forests (Peres et al., 2016; Wright et al., 2009).

China follows some of these patterns, but many aspects how China has changed are unique; they represent different challenges and most importantly different opportunities. For example, since 2000, China’s Gross Domestic Product has increased by approximately 270% and China is now the world’s largest economy (Ahrends et al., 2017). The economy has to support a population approaching 1.4 billion people (Deng et al., 2015; Wei & Ye, 2014). Both the economic and population growth have come at an environmental cost. It is estimated that China has lost between 1.9 and 2.7 million km${}^{2}$ of its original forest in the last 2 000 years (based on models of habitat suitability; Ahrends et al., 2017); this is an area approximately the size of the Democratic Republic of Congo. This has cost China in terms of primate diversity and population size. At least three species have been extirpated from China (*Pygathrix nemaeus, Hylobates lar yunnanensis, Nomascus leucogenys*) and the Hainan (*Nomascus hainanus*) and Cao-vit gibbons (*Nomascus nasutus*) will almost certainly not see the turn of the century without very effective conservation action (Fan, 2017; Turvey et al., 2017). Gibbons were once found from Xi’an in central China east to Shanghai and all the way south to the border (Fan, 2017; Turvey et al., 2017; Zhou & Zhang, 2013); now they are isolated in a few forest fragments to the south. Even orangutans were found in southern China only 12 500 years ago (Husson et al., 2009; Steiper, 2006), but they are no longer found on mainland Asia. A recent analysis considering 22 of the 27 primate species in China (Fan & Ma, 2018) suggests that 15 of the species have less than 3 000 individuals and that 81% of the populations of all Chinese primates are declining (Estrada et al., 2017). These sorts of statistics clearly indicate that China has lost a great deal of ground in the battle to conserve its biodiversity, but they also illustrate that China has great potential in terms of primate conservation.

The objective of this opinion article for this special issue on Primates and Primatology in China is to evaluate one road for future conservation/development that could produce very positive gains for China’s primates; namely forest restoration. There is clearly the need for restoration, as many of China’s primates are only found in small isolated forests; thus expanding their habitat and connecting fragments is clearly vitally needed.

## MAKING RESTORATION A VIABLE CONSERVATION STRATEGY

For a large scale restoration project to be possible two conditions must be met. First, there must be the right societal conditions to make restoration possible and second the knowledge must be in hand to carry out such projects. In terms of the societal conditions, it appears that the timing is right for restoration projects. In November 1988, government of China enacted the "Law of the People’s Republic of China on the Protection of Wildlife" to facilitate the protection and management of wildlife. This law is the first truly comprehensive law to protect wildlife in China. However, since 1998, the Chinese government has enacted several national biodiversity conservation regulations, such as the Natural Forest Protection Project and Ecological Forest Compensation, which have been effective in improving environmental conditions in many areas (Ren et al., 2015; Xu et al., 2009). Government financing for protected areas has also increased following the launch of the Wildlife Conservation and Nature Reserve Construction Project and the Special Fund for Capacity Building of National-Level Nature Reserves. China is investing substantially in reforestation and tree planting efforts and this has totalled more than US$ 100 billion in the past decade alone (Ahrends et al., 2017; Li et al., 2013; Viña et al., 2016; Zhang et al., 2000; Zhang, 2015). China now has the world’s largest plantation area (approx. 800 000 km${}^{2}$ (approximately the size of Mozambique, Ahrends et al., 2017). At the same time, China is trying to reduce pressures on natural forests through strict bans on logging in primary forests and a massive expansion of its forest reserves to a current total more than 2 500 reserves covering 1.6 million km${}^{2}$ (this area includes 17.1% of the country, which is approximately the size of Iran or twice the state of Texas). Lastly, over the last two decades there has been a large movement of people from rural areas (i.e., next to the reserves where China’s primates are found) to the cities. In fact, the urbanization rate rose from 17.9 to 52.6% between1978 and 2012 and currently more than half of China’s population live in cities (Deng et al., 2015).

In terms of the right societal conditions to make restoration possible, an area still requiring a great deal of effort is that of hunting. In many areas of southern China, where forest cover is still substantial, primate populations can be dramatically reduced because of hunting (Harrison et al., 2016). Even though some primates have Class I Protected Animals in the Chinese Wildlife Conservation Law and hunting guns have been outlawed and confiscated, illegal hunting still frequently occurs as it has been a traditional practise that is promoted by poverty in local communities, the use of wildlife for medicinal uses, and poor knowledge and enforcement of the laws (Fan et al., 2014). For example, in south-west Guangxi Province, Francois’ langur (*Trachypithecus francoisi*) populations declined by 90% between the early 1980s and early 2000s, at which time the total population size was estimated to be only approximately 300 individuals found in 14 isolated populations. The researchers conducting the later survey concluded that the primary threat to the langur was hunting, primarily for traditional medicine (Li et al., 2007).

The second requirement is that the knowledge must be available to effectively carry out a large restoration projects. Globally, there are only a handful of studies about the response of primate communities to forest regeneration; however, these studies suggest that forests, and the primate community they support, can rebound very rapidly when left to recover or encouraged to recover. For example, Baya & Storch (2010) surveyed a site in Korup National Park, Cameroon that was abandoned 7–8 years previously and found populations of all eight species of diurnal primates that occur in the region; in addition, sighting frequency in this recovering area was not significantly different from other sectors of the park (Linder, 2008). In Kibale National Park, Uganda, seven years after an area of grassland was replanted with trees as part of a carbon offset program (Omeja et al., 2012), all species of diurnal primates were present in high numbers, including endangered red colobus and chimpanzee. Such studies give hope for the future.

Within China a great deal of research has accumulated over the last two or so decades that provides exactly the type of information needed for restoration/conservation efforts. To start such efforts accurate information on the state of the species to be targeted must be known. There is extensive survey information on the current size and threats to the country’s primates (e.g., Chen et al., 2015; Cui et al., 2016). The synthesis of this information will be vital in determining the locations to be prioritized in restoration efforts; however, other local aspects, such as the willingness of the local population to participate in restoration and to not hunt primates must also be considered. Next, information must be available on the habitat requirements of the species targeted for help from a forest restoration project and again, there are many detailed projects focusing on primate habitat use (Fan et al., 2009, 2012; Guo et al., 2008; Li & Rogers, 2006; Liu et al., 2013). Of particular importance if the restoration project is to involve active replanting of trees is detailed information on the diet of the primates. With this information, either the food trees of the animals can be planted, or species with similar nutritional traits can be used in the restoration effort. There have been a number of high quality studies done on the nutritional ecology of China’s primates (e.g., Liu et al., 2013; Ma et al., 2017; Hou et al., 2018). To make restorations efforts more effective, it is critical to understand the severity and geographical nature of threats to primates in China other than habitat loss (e.g., live capture for trade, bushmeat, and tourism; Li et al., 2003; Xia et al., 2016; Yang et al., 2007; Zhu et al., 2013). In addition, information on conservation genetics is needed to determine the nature of corridors that can allow population mixing (Liu et al., 2015). With respect to conservation genetics, information concerning past interpopulational gene flow and landscape barriers on both short (e.g., satellite and aerial photography) and long time frames (e.g., river barriers), while rare (but see Wang et al., 2017), will be particularly valuable in determining the dispersal capabilities of primates relative to different types of barriers and can be used to provide guidance as to the nature of corridors that can be constructed to facilitate population mixing (Guo et al., 2010; Wang et al., 2015, 2017).

## WHAT REMAINS TO BE DONE FOR RESTORATION IN CHINA

While this existing information is extremely valuable, what is lacking is data specific to restoration efforts and studies documenting behavioral patterns and responses of primates to the regeneration of forests. For restoration projects to be most effective, information on the survival strategies used in regenerating forests of all the major primate groups, including prosimians, macaques, colobines, and gibbons will be vital. While this is a formidable task, it also represents an exciting challenge to academics and Universities; one where the value of research can be illustrated to the government and public.

What now remains to be done is to pull this societal potential and information together to facilitate large-scale forest restoration efforts that is critically needed for primate conservation. Since so many primate species in China are only hanging on as small remnant populations that are often only composed of a few groups (e.g., the cao vit gibbon population is estimated to only involve 18 groups occupying forest patches of only 2 000 hm^2^ with only 3–4 groups in China (Fan et al., 2011)) the only way to effectively promote conservation of these primates in the wild is through restoration. This will likely require some sort of well financed coordinating agency that is able to rally national and international scholars, conservation organizations, government agencies, and the public to first provided the needed scientific information and then to use this information to promote both natural regeneration and reforestation efforts on a very large scale.

## CONCLUSION

Primates are very charismatic and are generally liked by both the Chinese people and international groups, thus they can act as “Flagship” or “Guardian Angel” species to promote the value of restoring these lands to a native forest (Bicca-Marques & De Freitas, 2010; Simberloff, 1998). It is my opinion that this is an exciting time to integrate restoration into conservation strategies to make informed and effective conservation and management decisions for the primates of China.
